# Paramedic perceptions of barriers and facilitators to the use of ambulance service appropriate care-referral pathways in Northern Ireland: a qualitative study

**DOI:** 10.29045/14784726.2024.12.9.3.13

**Published:** 2024-12-01

**Authors:** Karl Bloomer, Jamie Scott, Rebecca Smyth, Julia Wolfe

**Affiliations:** Northern Ireland Ambulance Service (NIAS) ORCID iD: https://orcid.org/0000-0002-7822-4528; Ulster University ORCID iD: https://orcid.org/0000-0003-2402-021X; Northern Ireland Social Care Council (NISCC) ORCID iD: https://orcid.org/0009-0003-0832-5088; Northern Ireland Ambulance Service (NIAS) ORCID iD: https://orcid.org/0000-0002-3104-8360

**Keywords:** ambulance, decision making, paramedic, referral

## Abstract

**Introduction:**

Paramedic clinical practice has seen significant evolution from the traditional role of transporting patients to an emergency department (ED). An evolving and flexible scope of practice, modernisation and healthcare reform has necessitated the development of a range of referral pathways for paramedics, with the aim of ensuring that service users receive the most appropriate care at the point of contact. Ambulance conveyance rates to EDs in Northern Ireland (NI) have only occasionally fallen below 75%. A study examining a Northern Ireland Ambulance Service (NIAS) referral pathway showed a much lower referral rate than those of comparable ambulance services. A similar study found that over 70% of people who experience a fall are not referred to falls prevention services. This study aimed to identify what paramedics perceive are the barriers and facilitators to the use of patient care pathways (PCPs) in NI.

**Methods:**

In this single-centre qualitative study, participants were recruited using volunteer sampling. Data were collected through 11 semi-structured interviews until data saturation was reached. Online interviews were recorded, transcribed verbatim and thematically analysed.

**Results:**

Five main themes were constructed during analysis. The participants discussed their perceptions of the barriers and facilitators to utilising PCPs in relation to risk, cultural issues, person-centred practice, inter-professional communication and operational infrastructure.

**Conclusion:**

The study provides insight into perceived barriers and facilitators to the use of PCPs, while indicating the existence of a paramedic workforce dedicated to achieving the best outcomes for people in their care. The themes identified are consistent with existing literature that calls for standardised pathways across regions. Future research should investigate the link between the NHS 111 service and ambulance demand. In order to facilitate the complex decision making involved in referrals, relevant knowledge and skills should be embedded in paramedic education. Efforts should be made to improve inter-professional communication and awareness of the paramedic scope of practice and knowledge base. An intervention designed to reassure staff who have concerns regarding clinical risk may improve referral rates.

## Introduction

Historically, paramedics had limited autonomy and were typically obliged to convey all patients, regardless of clinical need, to an emergency department (ED) (Halter et al., 2011; Scott, 2020). It is now widely recognised that not every patient who contacts the ambulance service requires ED conveyance, and it is suggested that up to 50% of those who call 999 do not require ED attendance (Keogh, 2013). Subsequently, ambulance services have now embedded the practice of safely managing patients in the community by utilising ‘see and treat’ and ‘see, treat and refer’ pathways (Blodgett et al., 2021). As such, the modern paramedic is expected to make complex decisions about patient care in a variety of circumstances, including frequent referral and discharge decisions (O’Hara et al., 2015; Reay et al., 2018).

Patient care pathways (PCPs) have been introduced to facilitate referrals to general practitioners (GPs), minor injury units (MIUs) or district nursing teams, for example. The option to refer to these PCPs ensures that people receive the most appropriate care in the right place at the right time (Association of Ambulance Chief Executives, 2020). Additionally, safe and effective use of these pathways may result in a reduction of ambulance utilisation and demand on EDs, meaning ambulances are more readily available for life-threatening emergencies (Health Foundation, n.d.).

A recent review noted that English ambulance services were increasing their ED non-conveyance rates through a range of pathways, discharge decisions and access to alternative destinations (Carter, 2018). From 2011/2012 to 2015/2016, rates of non-conveyance increased from 33.9% to 37.9%, with no rise in re-contact rates (NHS England & NHS Improvement, 2019). Furthermore, there is acceptance that a figure of 35% can be safely managed without the need for ED conveyance (O’Cathain et al., 2018). The Northern Ireland Ambulance Service (NIAS) discharged or referred 25% of patients using pathways (NIAS, 2020). Diabetic and falls-specific studies have shown lower referral rates in NIAS than comparable ambulance services (Bloomer, 2021; Scott, 2020; Snooks et al., 2017).

This study aimed to explore what paramedics perceive as barriers and facilitators in the use of these pathways.

## Methods

The consolidated criteria for reporting qualitative research (COREQ) guidelines have been used for the reporting of this study (Tong et al., 2007).

### Setting

NI is split into five Health and Social Care Trust (HSCT) areas for the purposes of healthcare provision. The NIAS HSCT is the sixth Trust in NI and provides emergency and urgent care services throughout all five of these geographical areas, serving a population of over 1.9 million (Northern Ireland Statistics and Research Agency, 2022).

Historically, paramedics were trained vocationally in NI, under the Institute for Health and Care Development (IHCD) qualification. The Health and Care Professions Council (HCPC) now requires all newly trained paramedics to be educated to bachelor’s degree level. At the time of writing, the only bachelor’s degree course for paramedics in NI is the Paramedic Science BSc at Ulster University, which started in the academic year 2021‒2022, with its first graduates expected in late 2024 (NIAS, n.d.). Whereas an increasing number of paramedics in NI will possess a paramedic science degree if they have studied at a university outside of NI, the majority of NIAS paramedics are IHCD trained.

### Recruitment

Volunteer sampling was used, with participants asked to respond to a study invitation that was circulated Trust-wide via the NIAS Daily Bulletin announcement. Staff were able to self-select by replying to an email to register their interest. Information sheets were provided to each participant in advance of each interview, and informed consent was obtained prior to data collection. There were no incentives given and all participants took part in their own time.

### Data collection

This was a single-centre study of qualitative design. Data were collected through individual, online, semi-structured interviews with NIAS paramedics. A topic guide was developed and piloted with two participants by both KB and JW. These pilot interviews suggested no changes to the schedule and therefore were included in the data analysis. All interviews were conducted between May and October 2022, with a total of 11 participants interviewed before data saturation was reached. Data saturation was defined as the point at which no new ideas occurred that were pertinent to answering the research question. Two members of the research team (KB and JW) had regular discussions to address data saturation in line with ongoing data analysis. Once the point of saturation had been unanimously agreed, no further interviews were conducted. No participants refused to participate or dropped out of the study, and no repeat interviews were carried out.

The interviews were conducted online and were audibly recorded, with at least one member of the research team present (KB or JW). The duration of the interviews ranged from 16 minutes and 4 seconds to 61 minutes and 46 seconds. The mean interview duration time was 33 minutes and 13 seconds.

Two team members (KB and JW) kept reflexive journals to remain mindful of their influence on the research. The content of these journals was discussed by KB and JW at regular team meetings. The authors, KB and JW, were mindful of the impact their positions as Clinical Pathways Lead and Research Manager, respectively, might have had on participant responses.

### Data analysis

Data collection and data analysis occurred concurrently, with researchers immersing themselves in and familiarising themselves with the data. Interview recordings were transcribed verbatim by the research team, with transcripts being checked for transcription accuracy and to confirm full anonymisation. No transcripts were returned to participants for comment or correction.

Open coding was carried out in a systematic, line-by-line fashion and independently by two members of the team using NVivo 12 software (Lumivero, n.d.). The two team members then cross-checked and compared their independent coding against each other’s and discussed similarities and differences in order to enhance understanding of the data. Codes were renamed, combined and refined in an iterative manner throughout this process.

Transcripts were analysed using Braun and Clarke’s six-step framework for thematic analysis, with a reflexive approach being taken (Braun & Clarke, 2006).

In terms of orientation to the data, an inductive, data-driven perspective was utilised, with no a priori models or frameworks being used to direct concepts. The themes were constructed solely based on the data content. A semantic approach was taken when focusing on the meaning of participants’ words. This allowed themes to be readily identified, as researchers were looking at explicit meanings and not at what participants may have been implying. An experiential qualitative framework, as opposed to critical, was used to ensure the experiences of participants were captured, and an essential realist philosophical perspective was taken based on the beliefs of the researchers that an objective reality could be identified through what participants assumed to be real. Participants did not provide feedback on findings.

### Eligibility criteria

Participants were over 18 years of age, willing and able to give informed consent for participation, HCPC-registered paramedics, employed within NIAS for at least six months and working in patient-facing operational duties. Table 1 shows the demographic information of the participants.

**Table 1.** Participant demographic information.

Sex		Age (years)		Length of NIAS service (years)	
Male	8	26–35	5	1–5	5
Female	3	36–45	3	6–10	3
		46–55	3	11–15	3

## Results

Five main themes were constructed during analysis. The participants discussed their perceptions of the barriers and facilitators of utilising PCPs in relation to risk, cultural issues, person-centred care, inter-professional communication and operational infrastructure.

### Theme 1: risk

The concept of risk was discussed frequently by most participants. This included concern around the potential harm that may come to patients if staff made the wrong decision. Participants acknowledged that these concerns influenced their decision making. It was also recognised that at times this may be a subconscious concern, despite concluding that a referral to a PCP was the right decision for the patient. Confidence in managing the clinical risk involved in referral was discussed particularly in relation to the potential for the patient to deteriorate between the time of referral and the desired follow-up.

*If we’re not overly comfortable . . . with a particular condition and the potential for that condition worsening between me referring and this team arriving . . . I think clinicians become concerned about leaving those individuals unsupervised, as it were, for any period of time, because if a patient requires intervention of any degree, then . . . there’s ultimately the risk of deterioration.* (Participant 4)

*Or whether it’s, you know . . . the possibility of their deterioration or need [for] further hospital or acute care for their . . . respiratory problem that maybe it’s not actually a pathway that’s going to be used with any great regularity.* (Participant 3)

Over half of participants also discussed the risk in relation to themselves. This included the risk to their professional registration should an adverse event occur, but also, and more frequently, the risk of receiving complaints.

*And there is a fear, you know, a feeling, just I think even the simple complaints that come in . . . because it automatically goes to a Station Officer. And the Station Officer calls you in and then there’s meetings and there’s statements and . . . Going on [in] our minds . . . is someone going to come back to me and say, ‘Well, you knew that this patient had issues. You knew that you had to . . . why did you leave them?’* (Participant 1)

Some participants perceived the inclusion and exclusion criteria of some of the pathways to be too risk averse. They felt paramedics would be willing and confident in making referrals on a wider cohort of patients, despite the perceived risk of patient deterioration. For example, if the patient’s National Early Warning Score (NEWS) was higher than that allowed by the criteria, this led to a belief that the clinical risk of the patient was too high to manage in a community setting.

*The criteria is too restrictive compared to other services I’ve worked for before. They are too rigid, it’s too risk averse.* (Participant 6)

*I know originally there was a thing with NEWS scores being low, and once it went over a certain score, they really weren’t interested. But I’ve actually found that if you contact them, they will actually take a lot sicker patients than we think they will.* (Participant 11)

Conversely, some participants felt that the clinical criteria in some PCPs facilitated referral, rather than acting as a barrier, by providing clear guidelines for who could be referred and who could not. This reduced ambiguity and therefore the perceptions of clinical risk.

*I do think it gives a lot more elbow room for . . . maybe a sense that there’s a distinct pathway and if there’s criteria that’s very easy for us to follow . . .* (Participant 3)

### Theme 2: cultural issues

The concept of culture and the role it plays in facilitating or acting as a barrier was generated as a theme. Several participants alluded to the ambulance service being historically thought of as a transport service, with some discussing how this may impact on the utilisation of PCPs. It was felt by participants that a change in culture was required, from these old ways of thinking to a culture that empowers staff.

*There’s also . . . the cultural aspect of the ambulance service . . . of we are just a vehicle, we go and pick them up and take them to hospital. There’s that side of the cultural aspect you have to deal with.* (Participant 6)

Participants cited a lack of education around the PCPs as a barrier, with one participant highlighting that the change in educational requirements required for practice may have an impact on changing this culture. Some also alluded to a positive cultural shift that was already occurring and suggested that future PCP referral rates may increase as a result.

*It’s a culture thing . . . and I know that it’s getting much better and I genuinely believe that we’re in a progressive service now. I really think the BSc . . . is a silver bullet because I think those students are starting their career, they’re excited, they’re happy and they’re not going to want to just sit on the fence and collect a wage, they’re going to want to push on . . .* (Participant 7)

### Theme 3: person-centred care

The desire to bring about the best outcome for the patient was mentioned more frequently than any other theme. Overwhelmingly, participants wanted to see the most appropriate and holistic clinical outcome for individuals.

*The pathways, to me, have made a massive difference to patient care, and the patient care now is above and beyond . . .. We just need to be able to get more patients in, because they’re so good at doing what they do, if they could just take more.* (Participant 5)

*[The patients] may [have] been lying, fallen and lying on the floor, and […] are being accepted into these places now quite quickly rather than sitting outside A&E for hours and hours. It can only be good for the patient, it can only be a better outcome.* (Participant 11)

### Theme 4: inter-professional communication

Communication was identified as both a barrier and facilitator. Some participants recounted occasions where they had experienced communication challenges with the referral pathways, or other healthcare professionals (HCPs). This was perceived to be due to a lack of two-way professional discussion. It was believed that other HCPs were often reluctant to accept referrals, hesitant to take responsibility for a patient, were unaware of how the pathways were supposed to function or were unreachable for the purposes of facilitating a professional conversation.

*I would sometimes phone if [the patient] didn’t need to go to hospital, but they’re still really adamant. They don’t really take what you’ve said, or even if you give them a full assessment, history or your observations, they don’t really want to listen to you basically.* (Participant 8)

Conversely, several participants noted that pathways opened opportunities for communication with other HCPs, and that this facilitated shared decision making, potentially leading to better patient care.

*I made a GP referral for a gentleman who was very reluctant to travel to the hospital with a high clinical facility score . . . and I had asked for very specific things from the GP. And then, after discussion the GP was able to suggest particular medicines or prescriptions that the son could actually go to retrieve from pharmacy. That worked. That worked well actually.* (Participant 3)

Participants also identified the ease of communication with staff in emergency ambulance control (EAC) as a positive factor influencing referral making. This only applied to those referrals made via EAC, rather than directly with the receiving pathways service.

*It seems to work relatively well. Communication between us and the control room staff that are taking the information in relation to a particular pathway, it does seem to be pretty fluent, you know.* (Participant 4)

Another theme that was created from the data involved the idea of failed referrals. Participants discussed how some pathways had a higher incidence of rejections. This was perceived to be influenced by the receiving clinician, and it was noted that rejected referrals may influence future decision making.

*Things . . . are quite frustrating. So there seems to be human factors involved in whether or not they’re going to accept, and there doesn’t seem to be much adherence to the actual acceptance criteria. So, it can be literally . . ., ‘no, no, we don't deal with that’. Everyone’s like, ‘Oh, you need to go to the [name of unit] with that’. ‘No, I don’t, I’m looking at your criteria right now and they meet it’.* (Participant 9)

### Theme 5: operational infrastructure

Participants highlighted that operational barriers to accessing pathways added to the complexity of decision making. Pathway variation and availability across the five different HSCTs, NIAS divisions and the geographical areas covered by NIAS were perceived as a challenge, and participants cited a need for universal pathways. This was a particularly potent problem when paramedics were working outside their normal area of operation.

*I know the pathways really well because I use them all the time. But when you’re obviously sent to a call out of division . . . you’ve to try and figure out if the same care pathway applies, or if they’ve a different system.* (Participant 8)

One participant, who previously worked in an area where the NHS 111 service was operational, commented that it seemed to create additional demand for ambulances and that calls originating from the 111 service tended to be for less acute presentations.

*111 was a big issue, just in my experience, we would spend an entire shift just cleaning up, you know, or doing 111 calls. And so when I came over here and we didn’t have 111, I definitely found that we were going to more appropriately ill patients, appropriately categorised patients . . . So in terms of utilisation of care pathways, while there were a lot more in England that I could utilise, over here patients are, I find, a bit sicker.* (Participant 7)

Some participants who had experience of clinical practice in other ambulance services mentioned that the current scope of practice of NIAS paramedics limited their referral options. It was felt that a lack of advanced urgent care clinicians within the service meant that patients that could have been managed at home by the ambulance service were instead reliant on referral to other HSCTs.

*Not one of our staff deals with blocked catheters, which is … very frustrating. In other services, they have primary care paramedics who can deal with all of that. They can prescribe meds. So if I was out in the car and I had a patient with a blocked catheter, the primary care paramedic would have sorted that.* (Participant 6)

## Discussion

This study highlights several barriers and facilitators to paramedic referral to PCPs in NI. Five key themes were constructed from the data, with participants discussing several concurrently. This highlights a degree of interdependency between themes and presents a complicated picture for interpretation.

There was a perception among some participants that clinical guidance from the HSCTs, as well as the parameters of some referral pathways, were risk averse in nature. This was thought to lead to lower referral rates, with paramedics taking patients to ED by default, despite concluding they were suitable for referral. Conversely, some participants argued that the clinical criteria in some referral pathways actually empowered them to refer. Although there is no clear consensus on this theme, it highlights the importance of careful design and wording of referral pathways, particularly inclusion and exclusion criteria.

Participants identified the presence of conflicting cultures when it came to referral. More recently qualified paramedics appeared more confident in the referral aspect of their role than more experienced paramedics. This confidence in referring may be due to the recent changes in how paramedics are educated, with level of education being linked to confidence in decision making (Sheffield et al., 2016). However, further research is required to investigate any underlying link. 

Concerns were raised surrounding complaints and adverse incidents and how these would be handled by management and/or the HCPC. Participants believed that even appropriate clinical decisions may be viewed negatively. This supports previous research that highlighted an element of self-protection in paramedic clinical decision making (Porter et al., 2008; Scott, 2020) and ties in with the theme of risk that was generated during analysis.

The finding that inter-professional communication is perceived as a barrier to referral is in line with previous research by O’Hara et al. (2015) and Reay et al. (2018), with Reay et al. (2018) also identifying a feeling that paramedics’ professional knowledge was undervalued by other HCPs.

Participants also cited wider issues with system infrastructure and services that were available in one geographic location or within one HSCT but not another. Participants described a system where pathways existed at limited times ‒ what would be viewed as traditional primary care hours ‒ and reverted to a culture of ED and in-patient care only when outside of these hours. The need to shift the system to reflect modern healthcare has been accepted for some time (Bengoa et al., 2016). Furthermore, the findings highlight variance among clinicians receiving paramedic referrals, with some even appearing perplexed that paramedics were referring to them. The likelihood of referrals being accepted to established ambulance pathways was reported to be, at times, dependant on who the receiving clinician was. Participants claimed that they could predict the referral outcome even before a clinical conversation took place. There are many potential explanations for this, including varying appropriateness of paramedic referrals (Burns, 2018) and poor communication skills, which have previously been linked to healthcare outcomes (Ang et al., 2013).

Participants that had experience working in other ambulance services raised interesting issues that, while relevant, fell somewhat outside the scope of the current study. The current limited scope of paramedic practice in NI was seen as a barrier to the ambulance service managing patients in the community, instead relying on referral to other HSCTs.

In addition, the lack of an NHS 111 service in NI may have an impact on referral rates, as participants who had previously worked in areas where this service was operational noted that many of their calls were from 111, were less acute and were thus more suitable for referral. However, the lack of a 111 service in NI may lead these same patients to call 999, making this a difficult presumption to evidence. Future research is needed to investigate any link between the absence of a 111 service in NI and its impact on ambulance service demand in the region.

Pathway variation and availability across the different HSCTs were identified as a barrier, supporting similar findings by O’Hara et al. (2015), who further identified that conveyance to ED was often used as the ‘default’ option to reduce the risk of delays to treatment or of leaving patients unsupported.

### Limitations

It is important to consider that this study was conducted at one moment in time and within a set time period. Further research is needed to obtain a dynamic insight into the potential changing perceptions of barriers and facilitators to PCPs among paramedics.

Volunteer bias is a generally accepted phenomenon (Jordan et al., 2013), and this study derived its data from a volunteer sample; as such, caution should be used when generalising the findings to the wider paramedic profession outside of the context of NI.

Both interviewers (KB and JW, both HCPC-registered paramedics) were known to participants within the organisation at the time of data collection as the Clinical Pathways Lead and Research Manager, respectively. However, neither researcher held a direct managerial role over any of the participants, and both researchers were new to these posts within the eight weeks prior to data collection commencing. All paramedics involved were encouraged to be honest in the reporting of their experiences of PCPs and were made aware that this study was being conducted in order to improve referral pathways in NI. However, it should be acknowledged that the identities of the researchers conducting the interviews may have influenced results.

In this study, data saturation was used as an end point for data collection. Although this served as a pragmatic tool, upon reflection, the authors of this article acknowledge that, as ‘analysis can never be complete’ in reflexive thematic analysis, data saturation is not a concept they would strive to use again in this type of study (Braun & Clarke, 2019). Instead, an interpretive judgement would be made by the research team regarding when to stop data collection, coding or theme generation, with a focus on the quality of the data collected over data quantity.

## Conclusions

The authors believe that the study’s findings demonstrate a profession determined to get the best and most clinically appropriate outcomes for people who call on their services. Frustration was evident in the data where clinically appropriate referrals could not be completed due to perceived barriers. The study provides an insight into some of the perceived barriers and facilitators to the use of PCPs within one ambulance service. The themes identified by participants in this article are consistent with existing literature; however, there are likely to be additional barriers and facilitators influencing the utilisation of PCPs in other contexts, which could be explored in further studies.

Education around PCPs and promotion of their use is embedded within paramedic science degree programmes, with some participants alluding to a positive cultural shift occurring as a result. It remains to be seen if the change in educational requirements has a significant impact on changing culture within ambulance services. Prospective cohort studies comparing referral rates between degree-educated paramedics and their IHCD-qualified counterparts would allow this to be tested.

The disparate nature of the availability of PCPs and their criteria/willingness to accept referrals across the five Trust areas in Northern Ireland arose as a barrier to their use. Efforts to address this have already been made, with details of available pathways and their criteria readily available to each clinician. Moves to standardise and centralise PCPs at a regional level may improve their use. 

Future research could also investigate the impact of the absence of an NHS 111 service in NI on ambulance demand and the types of calls paramedics attend. It is anticipated that the complexity of decision making within the paramedic profession is set to increase as more treatment and referral options come into existence. Therefore, appropriate training and skills should be embedded in paramedic education, as well as within the existing workforce, to facilitate the provision of person-centred care for the NI population.

## Acknowledgements

The authors thank the participants for giving up their time contributing to this research.

## Author contributions

KB and JW were responsible for the study conception and design, recruitment, consent process, data collection, transcription, coding, thematic analysis, interpretation of findings and drafting and amending the article. RS was responsible for study design, transcription, coding, thematic analysis, interpretation of findings and drafting and amending the article. JS was responsible for data analysis, interpretation of findings, drafting and amending the article. KB acts as the guarantor for this article.

## Conflict of interest

None declared.

## Ethics

Ethical approval was not required, as this was a qualitative study involving interviewing NHS staff only. Governance was provided by the Northern Ireland Ambulance Service (NIAS) HSCT and Southern Health and Social Care Trust (SHSCT). IRAS no: 313512. This research was conducted according to the principles of the Declaration of Helsinki.

## Funding

None.
